# Machine Learning–Guided Detection of Malignancy of Lung Nodules With Molecular Imaging–Guided Surgery

**DOI:** 10.1001/jamanetworkopen.2025.51734

**Published:** 2026-01-13

**Authors:** Feredun Azari, Gregory T. Kennedy, Andrew Hanna, Austin Chang, Ashley Chang, Bilal Nadeem, Azra Din, Edward Delikatny, John Kucharczuk, Sardar Azari, Sunil Singhal

**Affiliations:** 1 Division of Thoracic Surgery, University of Pennsylvania Perelman School of Medicine, Philadelphia; 2 University of Pennsylvania Perelman School of Medicine, Philadelphia; 3 Department of Radiology, University of Pennsylvania Perelman School of Medicine, Philadelphia; 4 Heart, Vascular, and Thoracic Institute, Cleveland Clinic Foundation, Cleveland, Ohio; 5 Thoracic Surgery Department, Stanford University, Palo Alto, California

## Abstract

**Importance:**

Over 1 million pulmonary nodules are discovered each year in the US, and many of these undergo molecular imaging–guided surgery to obtain a diagnosis. Locating a small nodule and determining its malignant potential is technically challenging and is prone to human error.

**Objective:**

To demonstrate use of a machine learning (ML) algorithm with molecular imaging to analyze imaging data during lung cancer surgery to determine malignant potential of nodules.

**Design, Setting, and Participants:**

Data were retrospectively analyzed from a prospectively collected database. Between 2014 and 2021, patients at the hospital of the University of Pennsylvania with lung nodules were included in the study. Patients in the model development set were randomly allocated into training and validation sets in an 8:2 ratio. Data were analyzed from January 2014 and December 2021.

**Main Outcomes and Measures:**

Algorithmic tumor to background ratio (TBR) detection was implemented for individual images using Image Processing Toolkit. Developed nomogram and artificial intelligence (AI) image analyzer were combined as an optical biopsy algorithm and tested prospectively between 2021 and 2024.

**Results:**

A total of 322 patients with lung nodules were included in the study, of whom 279 had complete clinical data for data analysis (175 [62.7%] female). The nomograms and image segmentation technology were developed using a large database of IMI videos (1014 video sequences) and demonstrated an area under the curve of 0.865 to 0.893 for malignant nodule assessment. On multivariate logistic regression analysis, patient smoking history of greater than 5 pack-years (patient pack-years [PPY] >5), ex vivo back table TBR greater than 2.0, ex vivo bisected tumor lesions TBR greater than 2.4, and in situ (inside the chest) fluorescence were found to have statistically significant associations with malignancy on final pathology. Prospective testing in an independent set of 61 consecutive patients during IMI-guided cancer surgery demonstrated a sensitivity of 93.8%, specificity of 100%, positive predictive value of 100%, and negative predictive value of 71%. The study algorithm determined malignant potential of the lesion in less than 2 minutes (mean [SD], 1.8 [0.17] minutes) compared with a mean (SD) of 34 (11) minutes with frozen section analysis.

**Conclusion:**

In this cohort study of patients with indeterminate lung nodules, intraoperative imaging data analyzed by AI accurately determined if a nodule was malignant. This has the potential to improve the diagnostic challenges that occur at the time of surgery

## Key Points

**Question:**

Can a combination of machine learning–guided image recognition and molecular imaging technology optically biopsy lung nodules?

**Findings:**

This cohort study with 322 patients evaluates the use of machine learning–based algorithms in conjunction with robust nomograms during intraoperative molecular imaging (IMI)–guided lung cancer resections. Results suggest that the algorithm can identify and estimate malignant potential of an indeterminate lung nodule in a fast and efficient manner, with an area under the curve of 0.865 to 0.893 for malignant nodule assessment.

**Meaning:**

These findings suggest availability and implementation of artificial intelligence or machine learning–guided optical biopsy represents an area of new development in surgical oncology. The combination of computational processing power and IMI can serve as a useful adjunct for fast clinical decision-making intraoperatively.

## Teaser

This cohort study evaluates use of a machine learning algorithm with molecular imaging to analyze imaging data during lung cancer surgery to determine the malignant potential of nodules.

## Introduction

Currently, 1.7 million lung nodules are discovered each year, and approximately 250 000 of them will be lung cancer.^1^ A major challenge is the diagnosis of these indeterminate solitary pulmonary nodules (SPNs), which are defined as nodules less than 3 cm in diameter.^2^ A variety of diagnostic options are currently available for the diagnosis of SPN, such as needle biopsies, bronchoscopy, or surgery as a last resort.^3^ During diagnostic surgery, the criterion standard for intraoperative diagnosis is frozen section analysis. Frozen sectioning is the process of a pathologist rapidly analyzing the nodule once removed to guide the next surgical steps. Under most circumstances, this takes significant time, is prone to human errors, adds time under anesthesia, and comes with economic costs.^4,5,6,7,8^

One emerging technology that has the potential to circumvent the current challenges encountered in thoracic oncology is intraoperative molecular imaging (IMI). This involves infusing a targeted fluorochrome into the patient that specifically localizes to tumor nodules and emits fluorescence, which can be visually detected during real-time surgery.^9,10^ Recent clinical trials have shown significantly improved resection and occult lesion detection rates using this technology.^10,11,12^ IMI has also been successful in localizing small quantities of cancer disease and has had similar accuracy levels as histopathologic frozen section analysis.^9^

A key component of IMI is quantifying fluorescence emission intensity from the nodule and surrounding normal lung to calculate a tumor to background ratio (TBR). This is a common approach for many imaging technologies, such as positron emission tomography and single-photon emission computed tomography scanning. In optical imaging during surgery, fluorescence analyses are usually performed postsurgically without any standardized method of fluorescence emission quantification. Therefore, data are nonstandardized and can vary vastly across machines, surgeons, and institutions.

Therefore, reliable, efficient, and nonsubjective IMI-guided optical biopsy using artificial intelligence (AI) and machine learning (ML) is a natural solution to assist the surgeon in a reproducible, standardized, and objective way that has not been possible previously given the dynamic nature of real-time operative decision-making.^13^ To date, there has been a precedence of incorporating ML assisting algorithms into clinical practice.^14^ These algorithms, with the rapid improvements in computer processing, are already improving the efficiency and accuracy of diagnosis in nuclear medicine, dermatology, and histopathology.^15^ However, the majority of current applications of this powerful technology rely on the analysis of standardized static anatomic landmarks, single organ systems, or controlled histopathologic segments using human-selected and human-trained staining protocols.^16^ Incorporation into real-time dynamic clinical decision assistance, particularly in optical imaging, has not been investigated because of logistical difficulties due to small population samples, algorithmic interpretability across different demographic and geographic settings, and lack of prospective validation.^17^

In this study, the goal was to develop and test an ML-developed approach to tumor fluorescence and immediately calculate its malignant potential in real time. First, we developed a nomogram to estimate malignancy from the largest lung nodule database for IMI accrued over the 7-year period at our institution. We then trained the ML software algorithm using image segmentation analysis for reliable and efficient fluorescence quantification, eliminating observer bias. Finally, to assess its clinical performance and applicability, we prospectively validated our approach’s accuracy in determining whether a lung nodule is cancer or not during IMI-guided lung cancer surgery.

## Methods

The overall design of the study is depicted in eFigure 1 in Supplement 1. Appropriate authorization was obtained from the University of Pennsylvania institutional review board (IRB). Data were retrospectively analyzed from a prospectively collected database. Between 2014 and 2021, patients with lung nodules were included in the study. The study was conducted in a manner compliant with the Health Insurance Portability and Accountability Act (HIPAA), and all aspects of the study adhered to the tenets of the Declaration of Helsinki. Informed consent was obtained according to IRB requirements. Based on institutional practice patterns, preoperative tissue sampling and diagnosis were not mandatory. The videos from the surgery identifying the nodule, the IMI data, and the final pathology from the nodule were coalesced and results were subsequently analyzed prospectively between January 2021 and October 2024. Results reporting adhered to Strengthening the Reporting of Observational Studies in Epidemiology (STROBE) reporting guidelines where applicable.

### Specimen Analysis and TBR Calculation

The mean fluorescence intensity of the tumors and normal lung parenchyma were calculated from ImageJ version 1.53u (National Institutes of Health) and MATLAB version R2022a (MathWorks) software with a minimum of 1000 pixels included. The tumor-to-background ratio (TBR) was calculated for the specimens.

### Statistical Analysis

Patients in the model development set were randomly allocated into training and validation sets in an 8:2 ratio. To reduce selection bias, all benign samples were randomly divided into the training and validation sets using proportionate random sampling, and the process was repeated 250 times for cross-validation. There was no crossover between the training and validation sets.

Twenty-two clinicopathologic variables were used in development of the OptiDX algorithm using a multiple logistic regression model, and entropy balancing was performed for the covariates in the 2 sets (eTables 1-3 in Supplement 1). Statistically significant variables were selected for final nomogram development and in the validation set. Elastic net regularization was performed to determine the accuracy of the logistic regression model over the linear regression model where applicable. To reduce selection bias and find the best combination of clinical variables for estimating malignancy, models with the highest area under the curve (AUC) on receiver operating characteristic (ROC) analysis were used to test the validation set. Given the objective of developing a nomogram that can be reliably and efficiently used in a clinical setting, models with the simplest parameters were selected. To identify the model with the highest estimating ability, we selected nomograms with the highest AUC rather than relying on *P* values. Both parametric and nonparametric statistical analyses were performed using SPSS version 27 (IBM) and R studio version 4.4.2 (R Project for Statistical Computing). *P* values less than .05 were considered statistically significant.

## Results

### Development of the Algorithm From the SPN Cohort

Of 322 patients with indeterminate lung nodules, 279 were found to be suitable for this analysis (175 [62.7%] female). The patients were deemed to have high-risk nodules that met clinical criteria for potentially harboring malignancy. The cohort was randomly divided into a testing cohort for algorithm development and a validation cohort at an 8:2 ratio (eFigure 1, eTable 1 in Supplement 1). Descriptive statistics of both cohorts, including sex, final tumor pathology, tumor subtype, and tumor differentiation are displayed in eTable 1 in Supplement 1. A total of 58 of 279 (20.7%) had preoperative tissue sampling, with 26 (44.8%) being nondiagnostic, inconclusive, or not able to rule out malignancy.

**Figure 1.**
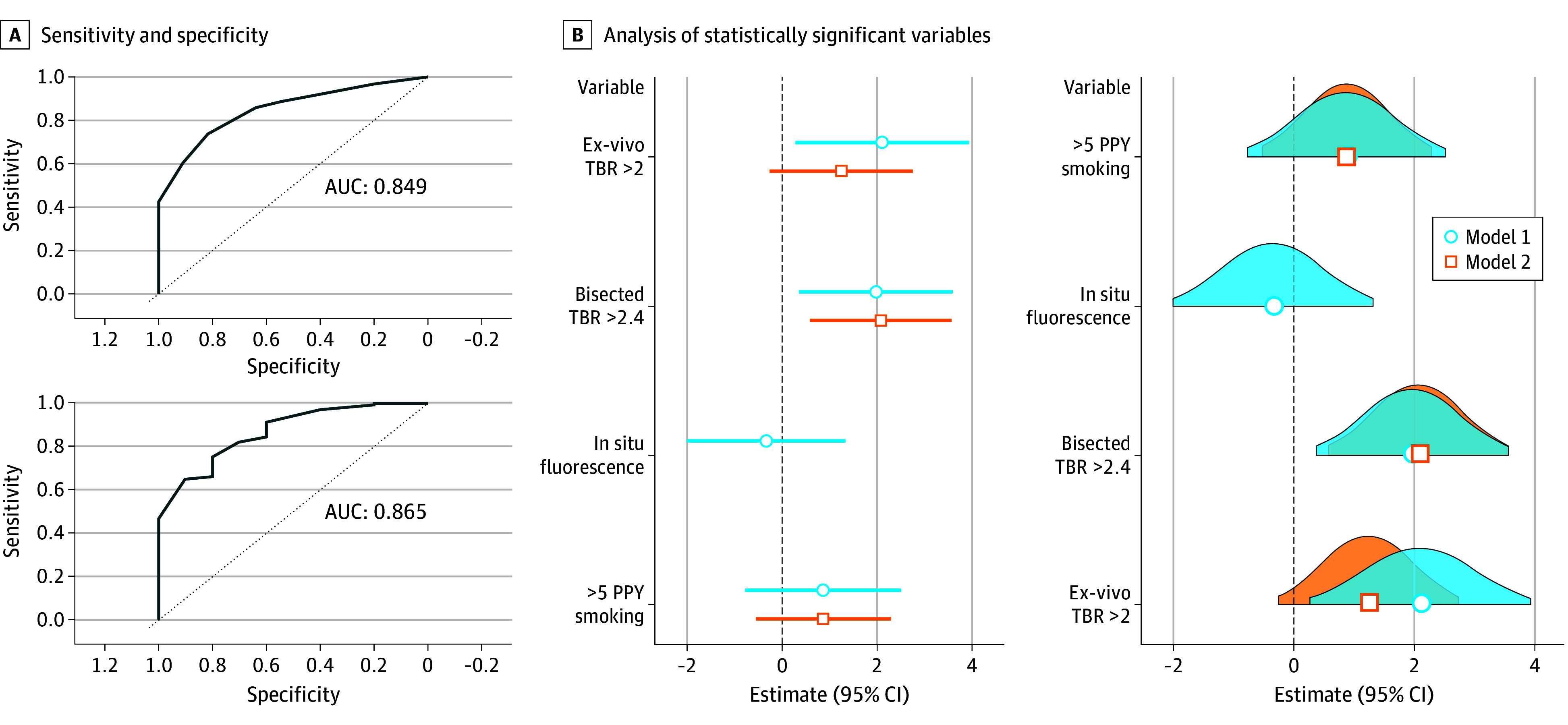
Receiver Operating Characteristic for Both Models A, Receiver operating characteristic generated for the 2 models; the top panel shows results for model 1 and the bottom panel for model 2. B, Analysis of the statistically significant variables in both models with their estimated outcome along with Gaussian distribution within the models. C, For binary predictors, green bars indicate yes (threshold met) and red bars indicate no (threshold not met). AUC indicates area under the curve; PPY, patient pack-years; TBR, tumor to background ratio.

**Figure 2.**
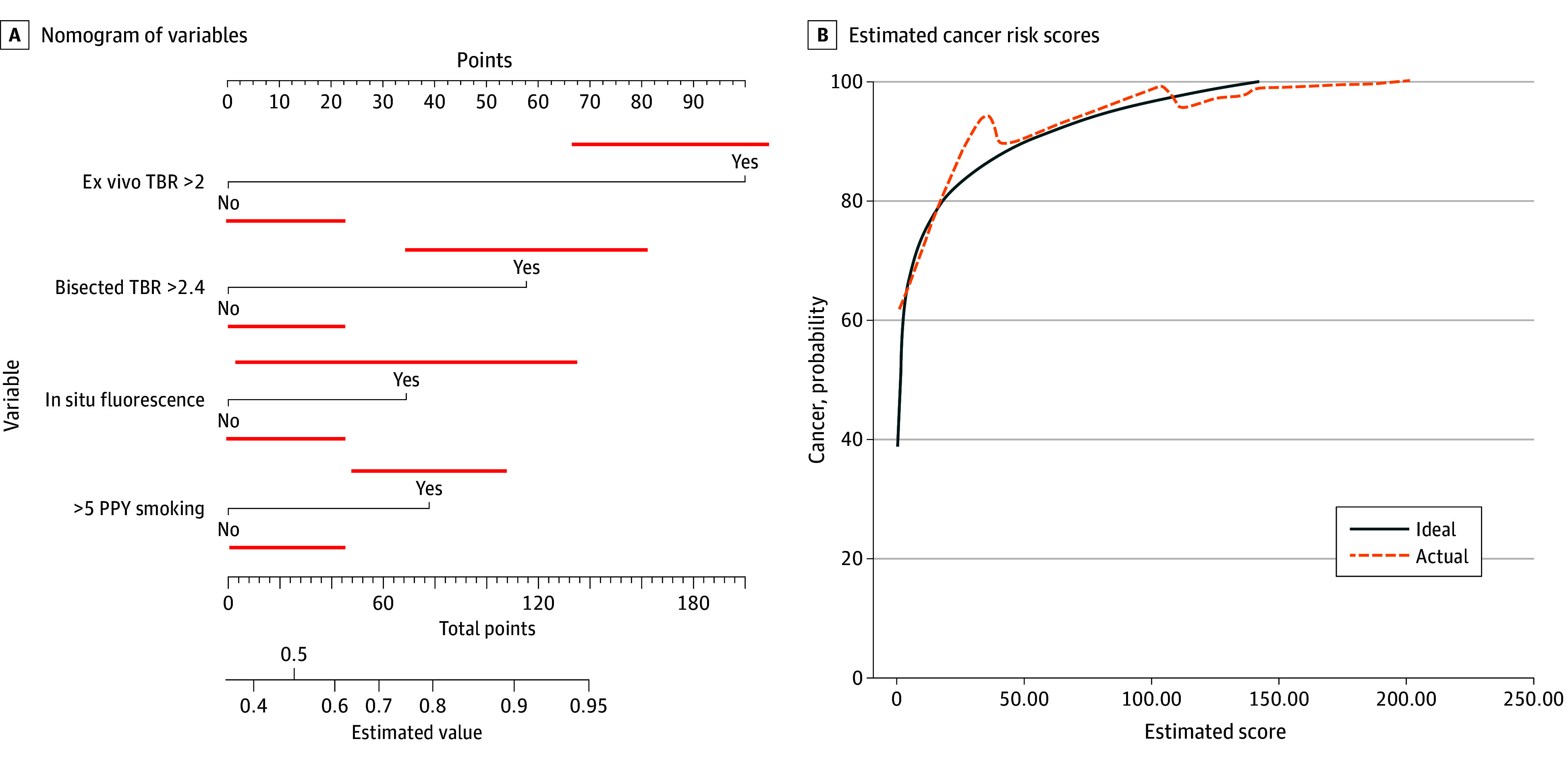
Nomogram and Logarithmic Distribution of Estimated Cancer Risk Scores A, Nomogram based on the statistical model. B, Logarithmic distribution of estimated cancer risk scores in the validation cohort. PPY indicates patient pack-years; TBR, tumor to background ratio.

Clinical, demographic, and histopathologic variables were analyzed for the 234 patients in the testing cohort as described in the Methods section. On multivariate logistic regression analysis, patient smoking history greater than 5 patient pack-years (PPY), ex vivo back table TBR greater than 2, ex vivo bisected tumor lesions TBR greater than 2.4, and in situ (inside the chest) fluorescence were found to have statistically significant correlations with malignancy on final pathology (eTable 2 in Supplement 1; Table 1). Poisson regression was performed using the aforementioned variables, which yielded 2 distinct models of association (Figure 1A and B). Two models were created due to the in-situ fluorescence variable having a lesser association with the estimation ability of the overall algorithm. However, given that in situ IMI-guided assessment is a key component in fluorescence-guided resection of nodules and slight improvement in AUC of the overall model, we elected to assess the estimation ability of in situ fluorescence with other variables in model 1 and removed it from statistically significant variables in model 2. In model 1, the back table nonwedge resection TBR measurement was found to be statistically significant (ex vivo TBR >2: OR, 13.30; 95% CI, 1.27-138.00; bisected nodule TBR >2.4: OR, 4.44; 95% CI, 0.76-26.10) (eTable 2 and eTable 3 in Supplement 1), and model 2 showed wedged ex vivo TBR measurement to be statistically significant (ex vivo TBR >2: OR, 3.51; 95% CI, 0.78-15.80; bisected nodule TBR >2.4: OR, 8.01; 95% CI, 1.81-35.50). ROC curves were performed for each model, with model 1 (Figures 1 and 2) showing an AUC of 0.893 and model 2 an AUC of 0.843. Removing nonstatistically significant variables from each model decreased the AUC to 0.849 and 0.865 for models 1 and 2, respectively. Therefore, these variables were not excluded from the final nomograms (Table 1).

**Table 1.** Multivariate Analysis of Model 1 and 2 Variables With Calculated Odds Ratios (ORs)

Characteristic	OR (95% CI)	*P* value
Model 1		
Intercept	0.52 (0.09-2.82)	.44
Ex vivo TBR >2	13.30 (1.27-138.00)	.03
Bisected nodule TBR >2.4	4.44 (0.76-26.10)	.03
In situ TBR >1.5	2.44 (0.36-16.70)	.37
>5 PPY smoking history	2.73 (0.48-15.50)	.26
Model 2		
Intercept	0.99 (0.26-3.70)	.99
Ex vivo TBR >2	3.51 (0.78-15.80)	.01
Bisected nodule TBR >2.4	8.01 (1.81-35.50)	.01
>5 PPY smoking history	2.41 (0.59-9.91)	.22

Abbreviations: PPY, patient pack-years; TBR, tumor to background ratio.

Nomograms based on the OR from each model were constructed for the testing cohort (Figure 1B, Figure 2A) based on thresholds identified on loglinear regression. Models were then assessed in the validation set of 45 patients from the retrospective internal dataset (Table 1, Figure 1B and 2B; and eFigure 2 in Supplement 1). The computed values for the validation cohort were not significantly different from those of the testing cohort, with a similar range of values in each cohort (eFigure 2 in Supplement 1). The probability of cancer based on our models showed excellent concordance in the validation cohort (Table 1 and Figure 2; eFigure 2 in Supplement 1).

### Machine Learning–Guided Image Segmentation and TBR Analysis

The goal was to develop a rapid nonbiased, accurate, and high-fidelity solution that would improve upon current standards. As shown in eFigure 3 in Supplement 1, each time an image needs to be analyzed, it would have to be exported at the conclusion of the case and calculated by hand on a remote computer device. To avoid this, 6172 near-infrared (NIR) images from 234 patients in the testing cohort were uploaded into MATLAB 2022b Simulink software (eFigure 6 in Supplement 1). A schematic of the computer processing images is shown in eFigure 6 and eFigure 4 in Supplement 1. The mean (SD) time for the TBR calculation of the ML algorithm was 1.4 (0.11) seconds. There was no statistically significant variation in TBR measurements by our algorithm and current standard methods (eFigure 4A and eFigure 7 in Supplement 1).

The algorithm was then prospectively evaluated for accuracy and relative concordance with current methods in 8 patients. Manual calculation (not including file export times) after the image was taken took a mean (SD) of 95 (33) seconds. Our algorithm had statistically shorter times for TBR calculation (eFigure 4 in Supplement 1).

### Integration of Nomogram and ML-Powered Image Segmentation

The final rendition of our algorithm included a combination of AI-based TBR calculation and a log-linear regression model to assess malignancy estimation capability in the validation cohort. Our algorithm recognizes the input image, calculates the necessary variables, and automatically inputs calculations into the model or nomogram, which then presents the surgeon with percentage risk of malignancy.

In the validation cohort, TBR calculations by the ML algorithm did not significantly differ compared with criterion standard TBR measurements manually (eFigure 4 in Supplement 1). However, our algorithm did have a smaller range of TBR (3.84) variation compared with manual calculation (6.17), likely due to operator bias in region selection. Our algorithm accurately calculated TBRs both for malignant and benign lesions, and these were not significantly different compared with the current standard (eFigure 4 in Supplement 1).

**Figure 3.**
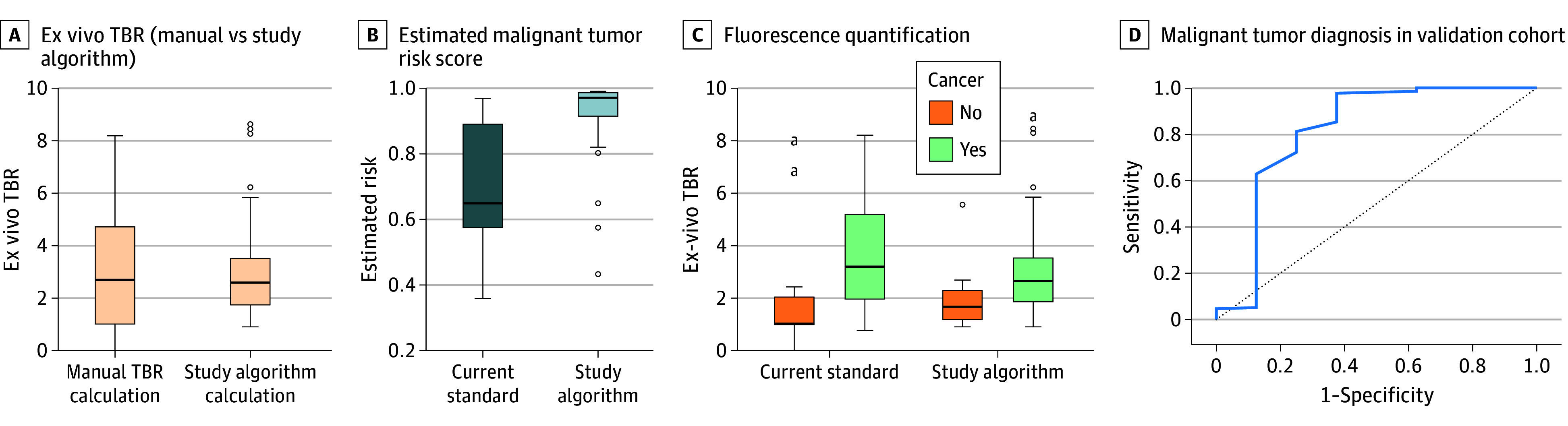
Comparison Between Methods in Validation Cohort A, In the validation cohort, all images were automatically entered into the device where it calculated ex vivo TBR values, which were then compared with manual mode. The box-whisker plot shows no difference between the groups. B, Fluorescence quantification between study algorithm (right) and the current standard (left) for malignant and benign pathologies with no significant difference observed in the box-whisker plot. C, Estimated malignancy risk scores for malignant and benign lesions by our algorithm in the validation cohort. D, Receiver operating characteristic curve of study algorithm estimating malignant risk in the validation cohort. Boxes indicate the interquartile range, horizontal lines within boxes indicate median values, whiskers indicate the range, and dots indicate outliers. TBR indicates tumor to background ratio. ^a^Estimated malignancy risk scores for malignant vs benign lesions in the validation cohort (solid line, malignant lesions; dashed line, benign lesions).

In terms of the identification of malignant potential based on our models, the ROC curve showed an excellent AUC of 0.864 (95% CI, 0.789-0.912) (Figure 3D). All 3 benign lesions in the validation cohort were identified by our algorithm, and 20 of 21 invasive adenocarcinomas (96%) were accurately classified as malignant. Subset analysis of the 1 missed invasive adenocarcinoma showed that upon bisection of the tissue, fluorescence was dampened by the presence of blood products in the image. Manual calculation also showed low tumor fluorescence due to autofluorescence produced by the blood products.

Cross-validation of our image analysis software incorporation into the nomogram in the validation cohort demonstrated that the algorithm had similar mean (SD) fluorescence quantification (TBR) compared with manual calculation but with significantly less variability and SD variance (2.78 [0.89] vs 2.89 [1.34]) (Figure 3A). The mean (SD) relative risk score for malignancy was higher for malignant lesions in the validation cohort than for benign lesions (0.98 [0.13] vs 0.61 [0.23]) (Figure 3C). The accuracy of the nomograms in estimating malignant vs benign lesions was the same for manual and ML-based fluorescence quantification (Figure 3B), with less variation in TBR calculation in the ML group for both cancerous and benign lesions.

Given that ex vivo and bisected tumor fluorescence quantification significantly contributed with nomogram accuracy, these 2 variables were assessed for their ability to estimate malignancy risk in both nomograms (eFigure 5 in Supplement 1). The results found that TBR elevation greater than 2 in ex vivo and 2.4 in bisected specimen analysis was associated with a high risk of harboring malignant lesions. Two benign nodules were misclassified as potentially malignant but had high TBRs (>8.5) and were found to be granulomas that falsely produced very high TBRs due to folate receptor β expression. This is a known phenomenon for pafolacianine-guided lung resections secondary to the overexpression of folate receptor β, and warning messages have been added to the algorithm for values higher than 8.5 TBR.

### Prospective Validation

Given the success of our algorithm in accurate fluorescence quantification and malignancy estimation, a prospective study of 61 continuous patients with 74 total pulmonary lesions undergoing pafolacianine-guided surgery was performed, and the results of our algorithm’s malignancy potential were compared with those of intraoperative frozen section histopathologic analysis (eFigure 6 in Supplement 1). A representative schematic of the study design is shown in Figure 3. Of the 64 malignant lesions, our algorithm identified 60 as potentially malignant (Table 2).

**Table 2.** Statistical Results From a Prospective Study of Study Algorithm for Malignancy Detection

Study algorithm outcome	Cancer (64)	Benign (10)	Total^a^
Malignant	60	0	60
Benign	4	10	14

^a^ Sensitivity, 93.8%; specificity, 100%; positive predictive value, 100%; negative predictive value, 71%.

The 4 patients in whom the diagnosis was missed had more than 60 pack-years of active smoking history with significant soot deposition in the lung parenchyma producing significant background parenchymal inflammation, making IMI a less suitable investigative option in these patients. There were 10 total benign lesions analyzed in the cohort, 4 of which were granulomas that produced a characteristically high TBR (>11), which in our prior analysis was found to be diagnostic of granulomas (eFigure 5 in Supplement 1). The other benign lesions were hamartomas and lymphoid aggregates. Our algorithm identified all 10 benign lesions (eFigure 5 in Supplement 1)

In our prospective cohort, our algorithm had a sensitivity of 93.8%, specificity of 100%, positive predictive value of 100%, and negative predictive value of 71% (Table 1). After prospective validation, the potential value of our algorithm and its use in conjunction with frozen section were explored. Our algorithm after image acquisition produced results on the malignant potential of the lesion in less than 2 minutes (mean [SD], 1.8 [0.2] minutes) compared with a mean (SD) of 34.0 (11.0) minutes with frozen section analysis.

## Discussion

IMI has the ability to complement and improve oncologic outcomes in thoracic surgery over the coming decades.^10,18^ As new targeted tracers progress through clinical trials and are being approved for clinical use, more surgeons can supplement their surgical decision-making with IMI, as it will allow the detection of subcentimeter primary nodules, the discovery of occult tumors, and the assessment of the efficacy of oncologic resection. However, as with all emerging technologies, IMI and its implementation are challenged by subjective biases and a lack of standardization.^19^ In this study, we proposed an optical biopsy device that incorporates statistically significant clinicopathologic variables in combination with an ML-trained image segmentation algorithm to estimate the likelihood of malignancy during IMI-guided resection of indeterminate solitary pulmonary nodules.

While IMI is a fairly new technology in the field of thoracic oncology, our dataset and experience with various NIR fluorochromes over the past decade is one of the most comprehensive in the literature.^9,11^ The cohort included patients with diverse demographic and clinical backgrounds (eTable 1 in Supplement 1). This allowed us to examine a significant number of variables, both clinicopathologic and intrinsic IMI-related parameters, to discover associations with malignant potential on final pathology. Our regression models demonstrated that fluorescence in situ, back table specimen fluorescence, bisected nodule fluorescence, and the presence of more than 5 PPY smoking were statistically significant variables during IMI-guided optical biopsies (eTable 2 in Supplement 1; Table 1, and Figures 1 and 2). Two distinct models with high AUCs were generated (0.849 for model 1 and 0.865 for model 2) (Figure 1A and B and Figure 2A). Strong AUC values are an integral component of any estimating nomograms, and one can argue is much more important than absolute *P* values in a clinical setting for each of the variables (eTable 2 in Supplement 1).^20^ High concordance in both the testing and validation cohorts of both models further highlights this statistical phenomenon (Figures 1, 2, and 3, and Table 1; eTable 2 in Supplement 1).

Given that TBR measurements are a key component in both algorithms, rapid, systematic, and standardized TBR calculations must be an integral component of our algorithm. Current methods for TBR and image analysis, as demonstrated in eFigure 3 in Supplement 1, are complex and are subject to significant investigator bias. Therefore, using sophisticated computer algorithms along with a large number of individual NIR images available in the testing cohort, we were able to design an algorithm component that performs image analysis using image segmentation in less than 2 seconds (eFigure 6 in Supplement 1). This ensures that each image obtained intraoperatively is analyzed in a predetermined manner and is not subject to observer bias (eAppendix in Supplement 1).

The combination of our algorithm with the AI-based image segmentation analysis for the final rendition of the optical biopsy device for testing in the validation cohort demonstrated encouraging results. Although there are published literature reports of the application of AI in surgical oncology, these studies often focus on static histopathologic and radiologic images without dynamic variables. In fact, our algorithm had a smaller TBR variation and STD than manual image analysis. The difference in TBR values did not detract from our algorithm’s ability to estimate malignancy potential in the validation cohort. The AUC of the ROC was 0.864, with all benign lesions and 20 of 21 invasive adenocarcinomas being identified accurately by our algorithm(Figure 3; eFigure 5 in Supplement 1). In the retrospective cohort, our algorithm allows accurate and efficient detection of malignant and benign lesions. The association between findings in the testing and validation sets demonstrated that both the nomogram and image analyzer can be used reliably to determine malignant potential. Our goal was to develop a device that immediately provided a malignancy risk score that the surgeon could combine with their clinical decision-making to become more efficient in the operating room.

While the results of retrospective analysis and validation were significant, we wanted to further confirm our hypothesis on the estimating ability of our algorithm in a prospective manner with 61 patients who were undergoing IMI-guided lung cancer resections (eFigure 6 in Supplement 1). The optical biopsy device in this realm showed excellent sensitivity and specificity (93.8% and 100%, respectively) (Table 2). The most concerning aspect was the 4 patients with cancer on final pathology that our algorithm characterized as negative. Close analysis of these patients demonstrated that three-quarters had a significant smoking history (>60 pack-years), which led to significant anthracosis secondary to light absorbing carbons (soot).^21^ The carbonaceous materials produce a characteristically black color to the lungs, and they absorb and emit light at all wavelengths. This causes significant false positive fluorescence in the normal lung parenchyma. Both the surgeon and our algorithm cannot decipher a difference in fluorescence from the tumor and the parenchyma, leading to false assumption that the tumor is not malignant. This is consistent with our previous known challenges in IMI interpretation in the presence of anthracosis.^22^

The ML algorithm offers additional value to thoracic surgeons who are looking to employ IMI-guided lung cancer resections. Our algorithm informs the surgeon about malignant potential in real time, which the surgeon can act on or use in conjunction with their clinical decision-making (eFigure 4 in Supplement 1). While ML software is not a replacement for histopathologic confirmation, it can allow the surgeon to perform other integral parts of an oncologic resection while the tissue confirmation is being performed by pathologists.^23^

### Limitations

There are several limitations that need to be accounted for in this study that we hope to address in the future. The nomogram, although validated internally for other solid organ malignant neoplasms, was developed using a lung cancer resection dataset with 1 possible optical contrast agent. The use of the nomogram in other IMI-guided cancer resections would need to be individually validated. In the current iteration, use of the technology outside of lung malignant neoplasms without folate receptor guided NIR fluorescence would not be recommend as there is lack of clinical studies with associated statistical analysis. Additionally, analysis of lymph nodes is also limited, which is inherent to pafolacianine and folate receptor–based technologies.

The image segmentation analyzer, while addressing many controversies of TBR utility in IMI, removes the surgeon from the decision-making process. In a clinical setting, expertise garnered over the years and the cognitive ability to synthesize comprehensive analysis of all other clinical data are indispensable components in oncologic surgery that are missed by AI algorithms. There are also several limitations that are inherent to IMI and optical tracer guided resections, which include presence of anthracosis, fluorescence dampening secondary to blood products, and false positive fluorescence by granulomas. Anthracotic lungs contain light absorbing carbons which absorb and emit light across a large range of light spectrum which creates false fluorescence across the lung parenchymal surface. In a patient with more than 60 PPY smoking history, there would limitations in use of this technology, which is evident in large subsections of patients with lung cancer.^22^ Blood products similarly act as a barrier in allowing the excitation and emission light to pass, therefore creating a conflict in highly inflamed cases where large products can be observed. Additionally, granulomas contain folate receptors and can have extremely high fluorescence, often with TBR values greater than 10; our device alerts the operator if this is a possibility but presents a limitation. Additionally, while, to our knowledge, our dataset and prospective validation are the most comprehensive in the field of IMI, external validation of our algorithm should be performed using multicenter randomized clinical trials in different NIR devices.

## Conclusions

In this cohort study of patients with indeterminate lung nodules, IMI in combination with AI was found to have the ability to reliably and rapidly identify malignant lesions during real-time lung cancer resections. The implementation of this technology can save health care costs, improve oncologic outcomes, and significantly decrease operative time in patient populations with marginal pulmonary reserves. The technology is considerably scalable and can be used across various open-source hardware and software.

## Notes

Supplement 1. eTable 1. Distribution of Demographic and Histopathologic Variables in the Testing and Validation Cohorts eTable 2. Statistical Analysis Showing the Logistic Regression Coefficient in Univariate Analysis for Models 1 and 2 eTable 3. Clinicopathological Variables Used in Initial Analysis eFigure 1. Pictorial Overview of the Study Design eFigure 2. Predicted Scores for the Nomograms Between the Testing and Validation Cohorts Demonstrate Similar Calculations That are Not Significantly Different eFigure 3. Current Method for Calculating TBR eFigure 4. Image Analysis eFigure 5. Modified Risk Score Distributions eFigure 6. Prospective Validation of OptiDx in IMI-Guided Lung Cancer Resections eFigure 7. Image Analysis Protocol eAppendix. eReference.

Supplement 2. Data Sharing Statement
