# Supplementary material for: Machine Learning–Guided Detection of Malignancy of Lung Nodules With Molecular Imaging–Guided Surgery
Source: JAMA Netw Open. 2026 Jan 13;9(1):e2551734. doi: 10.1001/jamanetworkopen.2025.51734 (PMC12801086; doi:10.1001/jamanetworkopen.2025.51734)
Supplement: Supplement 1. — eTable 1. Distribution of Demographic and Histopathologic Variables in the Testing and Validation Cohorts eTable 2. Statistical Analysis Showing the Logistic Regression Coefficient in Univariate Analysis for Models 1 and 2 eTable 3. Clinicopathological Variables Used in Initial Analysis eFigure 1. Pictorial Overview of the Study Design eFigure 2. Predicted Scores for the Nomograms Between the Testing and Validation Cohorts Demonstrate Similar Calculations That are Not Significantly Different eFigure 3. Current Method for Calculating TBR eFigure 4. Image Analysis eFigure 5. Modified Risk Score Distributions eFigure 6. Prospective Validation of OptiDx in IMI-Guided Lung Cancer Resections eFigure 7. Image Analysis Protocol eAppendix. eReference. [file jamanetwopen-e2551734-s001.pdf]

Supplemental Online Content

Azari F, Kennedy GT, Hanna A. Machine learning–guided detection of malignancy of lung nodules with molecular imaging–guided surgery. *JAMA Netw Open*. 2026;8(12):e2551734. doi:10.1001/jamanetworkopen.2025.51734

eTable 1. Distribution of Demographic and Histopathologic Variables in the Testing and Validation Cohorts
eTable 2. Statistical Analysis Showing the Logistic Regression Coefficient in Univariate Analysis for Models 1 and 2
eTable 3. Clinicopathological Variables Used in Initial Analysis
eFigure 1. Pictorial Overview of the Study Design
eFigure 2. Predicted Scores for the Nomograms Between the Testing and Validation Cohorts Demonstrate Similar Calculations That are Not Significantly Different
eFigure 3. Current Method for Calculating TBR
eFigure 4. Image Analysis
eFigure 5. Modified Risk Score Distributions
eFigure 6. Prospective Validation of OptiDx in IMI-Guided Lung Cancer Resections
eFigure 7. Image Analysis Protocol
eAppendix.
eReference.

This supplemental material has been provided by the authors to give readers additional information about their work.

© 2026 Azari F et al. *JAMA Network Open*.

Variable	Testing Cohort (n=234)	Validation Cohort (n=45)	Total (n=279)
**Gender**			
Male	82 (34.9)	22 (48.9)	104 (37.3)
Female	152 (64.7)	23 (51.1)	175 (62.7)
**Final Pathology**			
Benign	37 (15.7)	3 (6.7)	40 (14.3)
Invasive Adenocarcinoma	120 (51.1)	21 (46.7)	141 (50.5)
Neuroendocrine Tumor	1 (0.4)	1 (2.2)	2 (0.7)
Pulmonary Metastasis	24 (10.2)	9 (20.0)	33 (11.8)
Squamous Cell Cancer	12 (5.1)	3 (6.7)	15 (5.4)
Atypical Carcinoid	1 (0.4)	1 (2.2)	2 (0.7)
SCLC	6 (2.6)	-	6 (2.2)
Minimally Invasive Adenocarcinoma	15 (6.4)	2 (4.4)	17 (6.1)
Mucinous Adenocarcinoma	1 (0.4)	-	1 (0.4)
Adenosquamous	3 (1.3)	-	3 (1.1)
Smooth Muscle Tumor	1 (0.4)	-	1 (0.4)
Adenocarcinoma In-Situ	6 (2.6)	2 (4.4)	8 (2.9)
Typical Carcinoid	3 (1.3)	1 (2.2)	4 (1.4)
Other NSCLC	1 (0.4)	-	1 (0.4)
Adenoid Cystic Carcinoma	1 (0.4)	-	1 (0.4)
Mesotheliorma	2 (0.9)	-	2 (0.7)
Pleomorphic Carcinoma	-	1 (2.2)	1 (0.4)
Spindle Cell Tumor	-	1 (2.2)	1 (0.4)
**Tumor Subtype**			
Acinar	51 (21.7)	10 (22.2)	61 (21.9)
Lepidic	16 (6.8)	3 (6.7)	19 (6.8)
Papillary	22 (9.4)	7 (15.6)	29 (10.4)
Micropapillary	4 (1.7)	1 (2.2)	5 (1.8)
Mucinous	11 (4.7)	-	11 (3.9)
Solid	10 (4.3)	4 (8.9)	14 (5.0)
Basaloid	1 (0.4)	1 (2.2)	2 (0.7)
Keratinizing	4 (1.7)	1 (2.2)	5 (1.8)
Non-Mucinous	18 (7.7)	4 (8.9)	22 (7.9)
Colloid	1 (0.4)	-	1 (0.4)
Fetal	1 (0.4)	-	1 (0.4)
Non-Keratinizing	1 (0.4)	-	1 (0.4)

© 2026 Azari F et al. *JAMA Network Open*.

Variable	Testing Cohort (n=234)	Validation Cohort (n=45)	Total (n=279)
**Tumor Differentiation**			
Well-Differentiated	28 (11.9)	6 (13.3)	34 (12.2)
Moderately Differentiated	82 (34.9)	19 (42.2)	101 (36.2)
Poorly Differentiated	48 (20.4)	9 (20.0)	57 (20.4)
Undifferentiated	2 (0.9)	-	2 (0.7)

Data presented as No. (%). SCLC = small cell lung cancer; NSCLC = non-small cell lung cancer

eTable 1. Distribution of Demographic and Histopathologic Variables in the Testing and Validation Cohorts

	**Model 1**
	**Value (STD error)**
(Intercept)	-0.27 (0.93)
Ex-Vivo TBR	2.11 (0.93)
Bisected Nodule TBR	1.98 (0.82)
In-Situ Fluorescence TBR >1.5	-0.31 (0.85)
>5 PPY Smoking History	0.88 (0.83)
	**Model 2**
	**Value (STD error)**
(Intercept)	-0.01 (0.67)
Ex-Vivo TBR	1.25 (0.77)
Bisected Nodule TBR	2.08 (0.76)
>5 PPY Smoking History	0.88 (0.72)

eTable 2. Statistical Analysis Showing the Logistic Regression Coefficient in Univariate Analysis for Models 1 and 2

Supplementary Table for 22 Clinicopathological Variables Used in Nomogram Development

© 2026 Azari F et al. *JAMA Network Open*.

#	Variable Name	Category	Definition/Description	Included in Final Model
1	Age	Demographic	Patient age in years	No
2	Sex	Demographic	Male/Female	No
3	BMI	Demographic	Body mass index (kg/m^2)	No
4	Race/Ethnicity	Demographic	Patient racial/ethnic background	No
5	**PPY**	**Clinical History**	**Smoking history**	**Yes (Models 1 & 2)**
6	Current Smoking Status	Clinical History	Current/Former/Never smoker	No
7	Family History of Lung Cancer	Clinical History	First-degree relative with lung cancer	No
8	Prior Malignancy History	Clinical History	Previous cancer diagnosis	No
9	COPD	Clinical History	Chronic obstructive pulmonary disease	No
10	Occupational Exposure	Clinical History	Asbestos, silica, or other carcinogens	No
11	Nodule Size	Tumor Characteristics	Maximum diameter in centimeters	No
12	Nodule Location	Tumor Characteristics	Upper/Middle/Lower lobe	No
13	Nodule Morphology	Tumor Characteristics	Solid/Part-solid/Ground glass opacity	No
14	Spiculation	Tumor Characteristics	Presence of spiculated borders	No

© 2026 Azari F et al. *JAMA Network Open*.

#	Variable Name	Category	Definition/Description	Included in Final Model
15	Pleural Retraction	Tumor Characteristics	Pleural surface retraction	No
16	**In Situ Fluorescence**	**Imaging**	**TBR >1.5 during in-chest imaging**	**Yes (Model 1 only)**
17	**Ex Vivo TBR**	**Imaging**	**Back table specimen TBR >2.0**	**Yes (Models 1 & 2)**
18	**Bisected Tumor TBR**	**Imaging**	**Cut specimen TBR >2.4**	**Yes (Models 1 & 2)**
19	Fluorescence Uniformity	Imaging	Homogeneous vs heterogeneous uptake	No
20	Background Parenchymal Fluorescence	Imaging	Normal lung tissue fluorescence intensity	No
21	Resection Type	Surgical	Wedge/Segmentectomy/Lobectomy	No
22	Depth on CT scan	Radiological	Depth measured on CT scan from visceral surface	No

eTable 3. Clinicopathological Variables Used in Initial Analysis

© 2026 Azari F et al. *JAMA Network Open*.

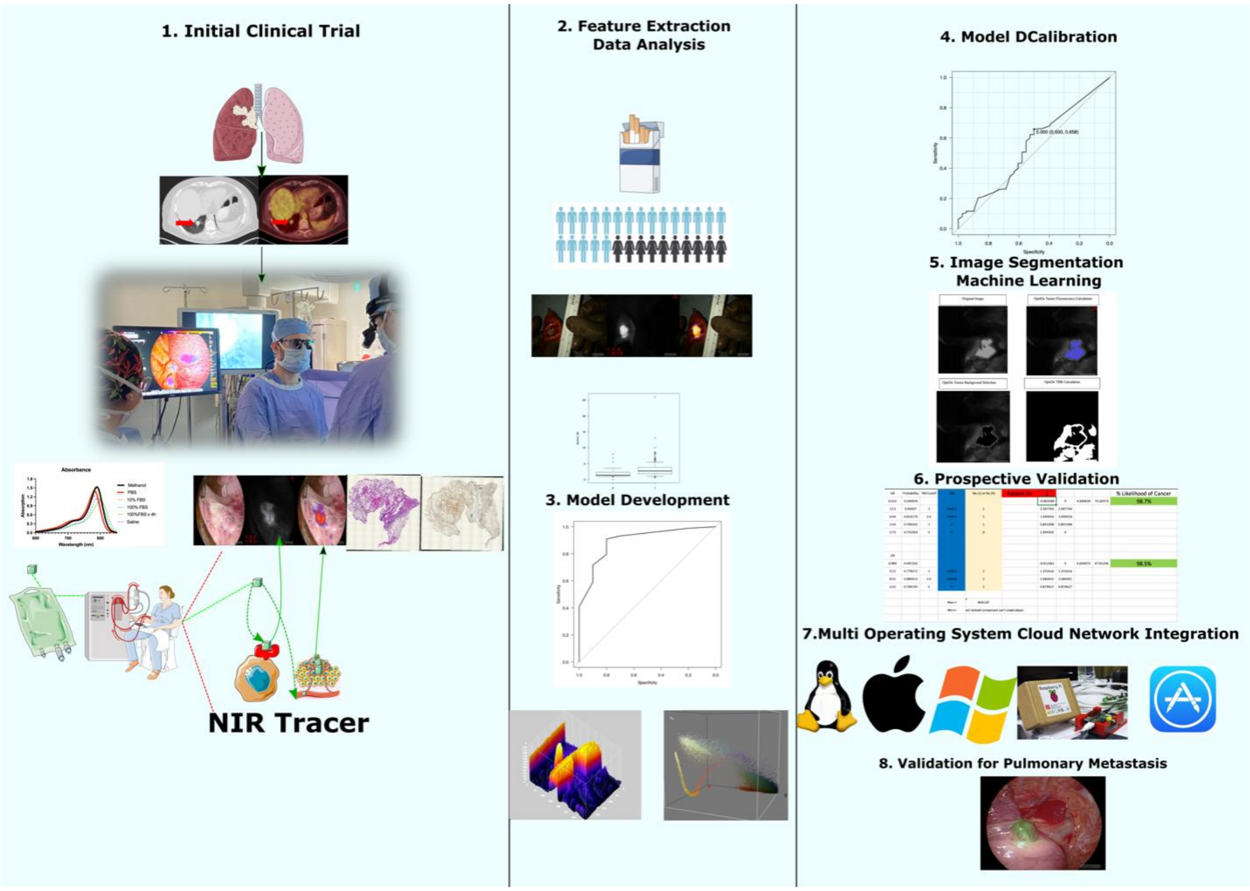

eFigure 1. Pictorial Overview of the Study Design

Left column depicts the clinical application of IMI in various clinical trials for lung cancer management. Middle column depicts retrospective analysis of >20 variables, including detailed image parameters and development of statistical models. Right Column: incorporating and validating the generated models using artificial intelligence in a prospective manner.

© 2026 Azari F et al. *JAMA Network Open*.

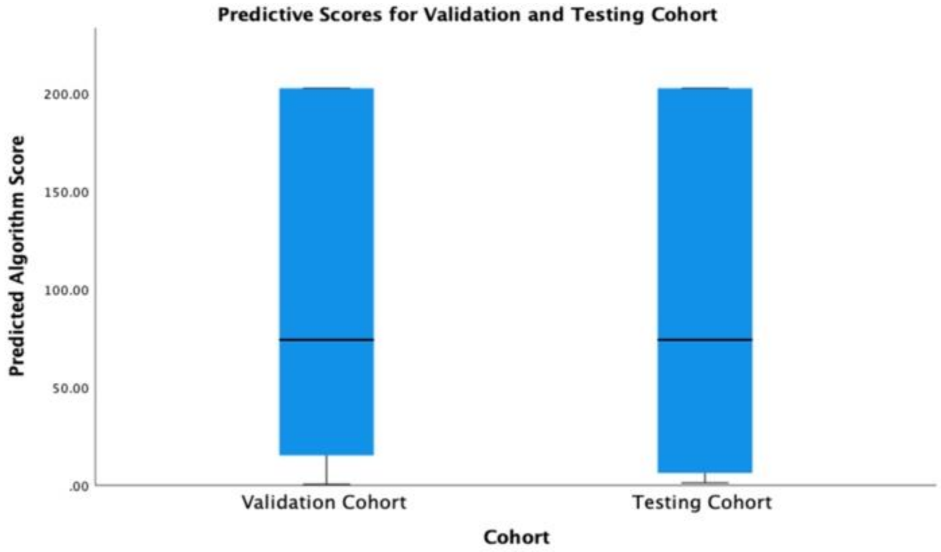

eFigure 2. Predicted Scores for the Nomograms Between the Testing and Validation Cohorts Demonstrate Similar Calculations That are Not Significantly Different

© 2026 Azari F et al. *JAMA Network Open*.

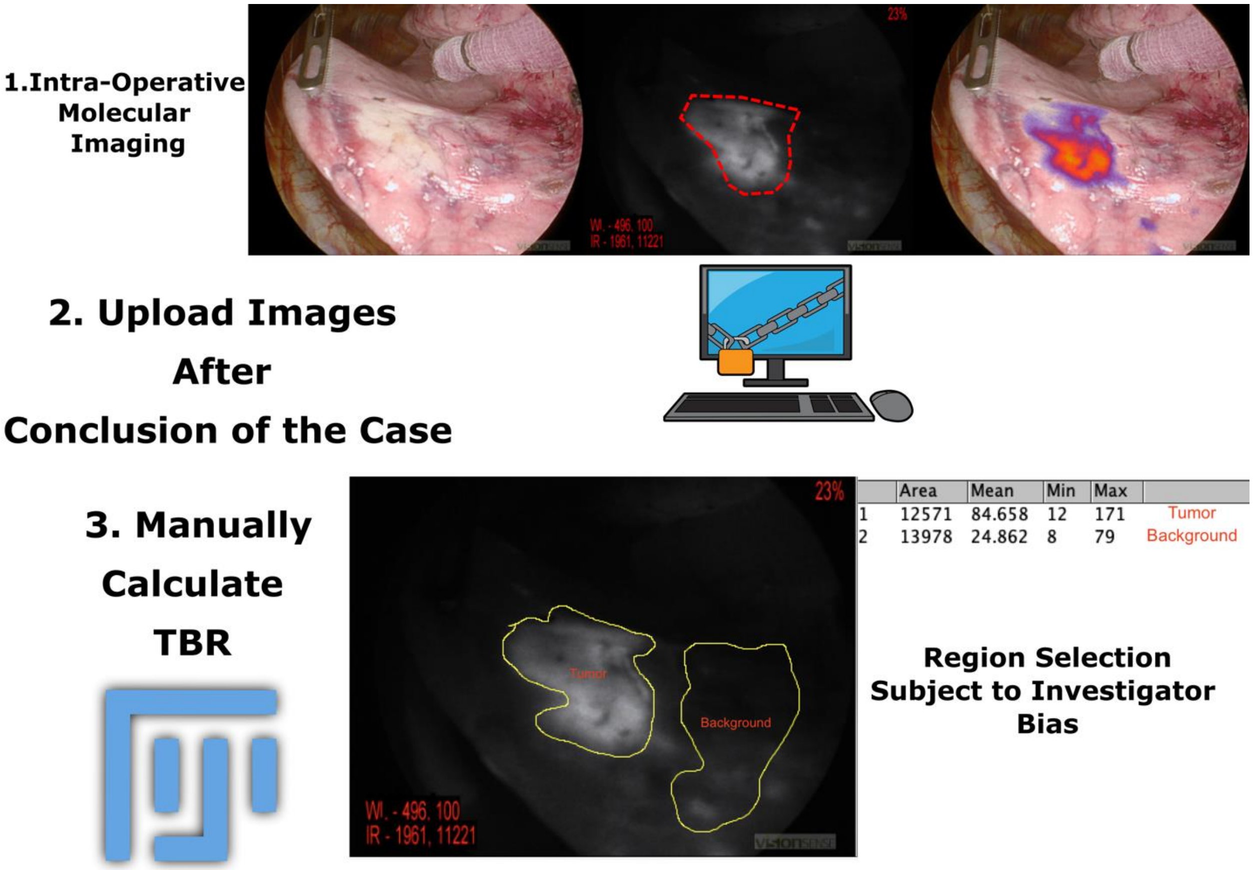

eFigure 3. Current Method for Calculating TBR
After the conclusion of the case, the researcher uploads the images and performs analysis using ImageJ. The region selection in this case is subject to researcher bias as they determine the appropriate area for Tumor and Background.

© 2026 Azari F et al. *JAMA Network Open*.

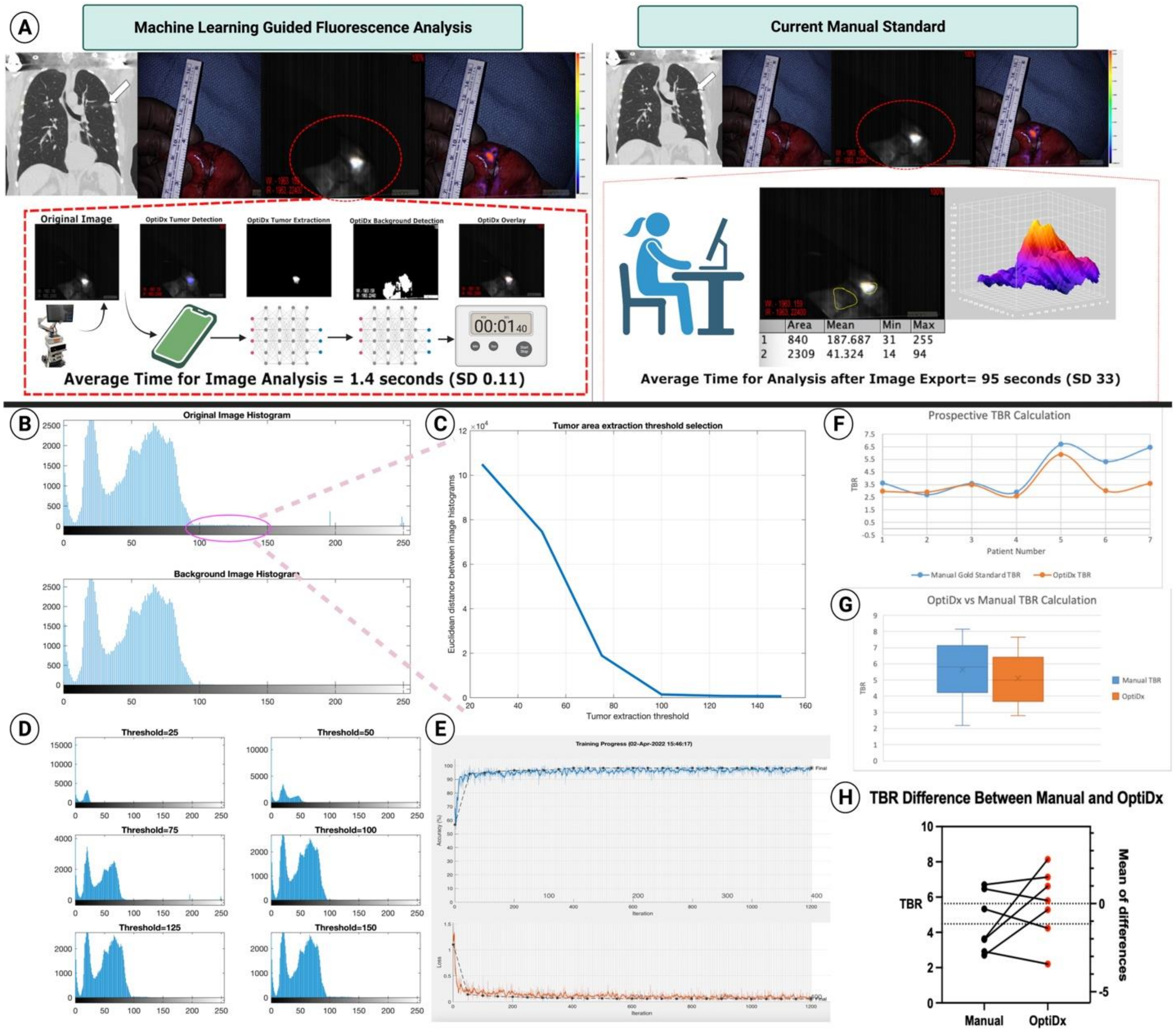

eFigure 4. Image Analysis

(A) *Left column*: Image analysis performed by OptiDx, where the computer algorithm identifies the area of interest and automatically extracts important parameters for TBR calculation. The process takes less than 2 seconds to complete. The results are then compared to pathology to determine if there is appropriate cellular internalization of the NIR tracer. *Right Column:* Conversely, as depicted in S-1, the current standard of fluorescence quantification requires image export and manual analysis at a remote location, which currently takes approximately a week. The process of quantification of appropriate regions (tumor and background) takes 90 seconds. **(B):** Raw fluorescence histogram of the tumor and the background with the *inset*

© 2026 Azari F et al. *JAMA Network Open*.

corresponding to fluorescence variation unique in segmented areas of the tumor nodule and not background. **(C)** The algorithm then applies incremental thresholding **(D)** and analyzes the Euclidian distances between tumor fluorescence areas to generate the highest TBR calculation. **(E)** The image analysis shows a high rate of image analysis (blue) and a low rate of data loss (orange) as the algorithm analyzes the image segments.

(F) Prospective validation of the image fluorescence analysis component in subsequent patients demonstrates similar TBR calculation between OptiDx and the current manual extraction method (gold standard). **(G):** Box and whisker plots show that there is no statistically significant variation between the OptiDx and manual TBR calculations in the validation cohort. Similarly, prospective analysis of OptidDx **(H)** showed no significant variation between manual calculation and OptiDx.

© 2026 Azari F et al. *JAMA Network Open*.

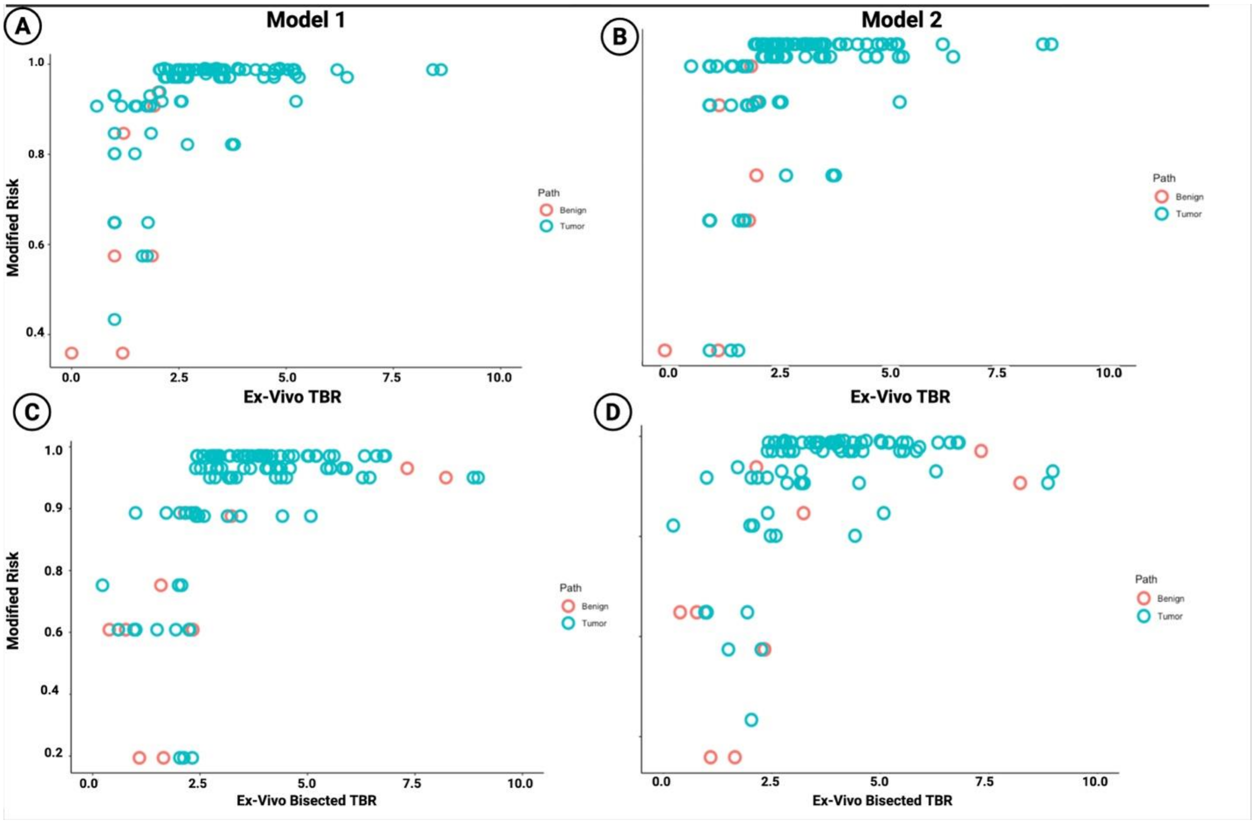

eFigure 5. Modified Risk Score Distributions

(A-B) Modified risk score distribution based on ex vivo specimen analysis using OptiDx for Model 1 (left) and Model 2 (right). **(C-D)**: Modified risk score distribution of malignant and benign lesions based on bisected specimen analysis using OptiDx for Model 1 (left) and Model 2 (right).

© 2026 Azari F et al. *JAMA Network Open*.

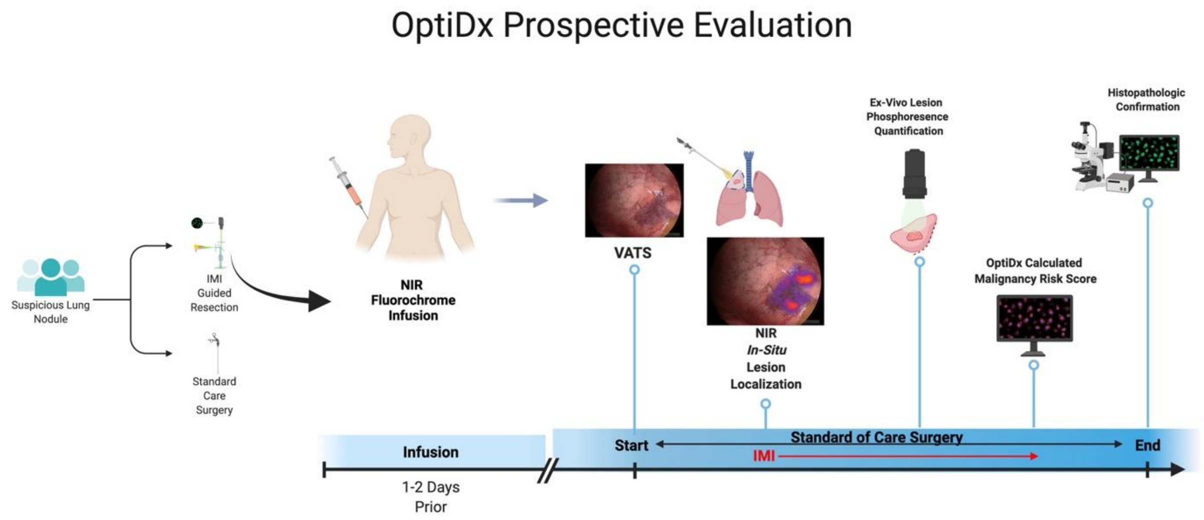

eFigure 6. Prospective Validation of OptiDx in IMI-Guided Lung Cancer Resections

Patients with concerning or biopsy-proven IMI were enrolled in pafolacianine- based on trial inclusion and exclusion criteria.

© 2026 Azari F et al. *JAMA Network Open*.

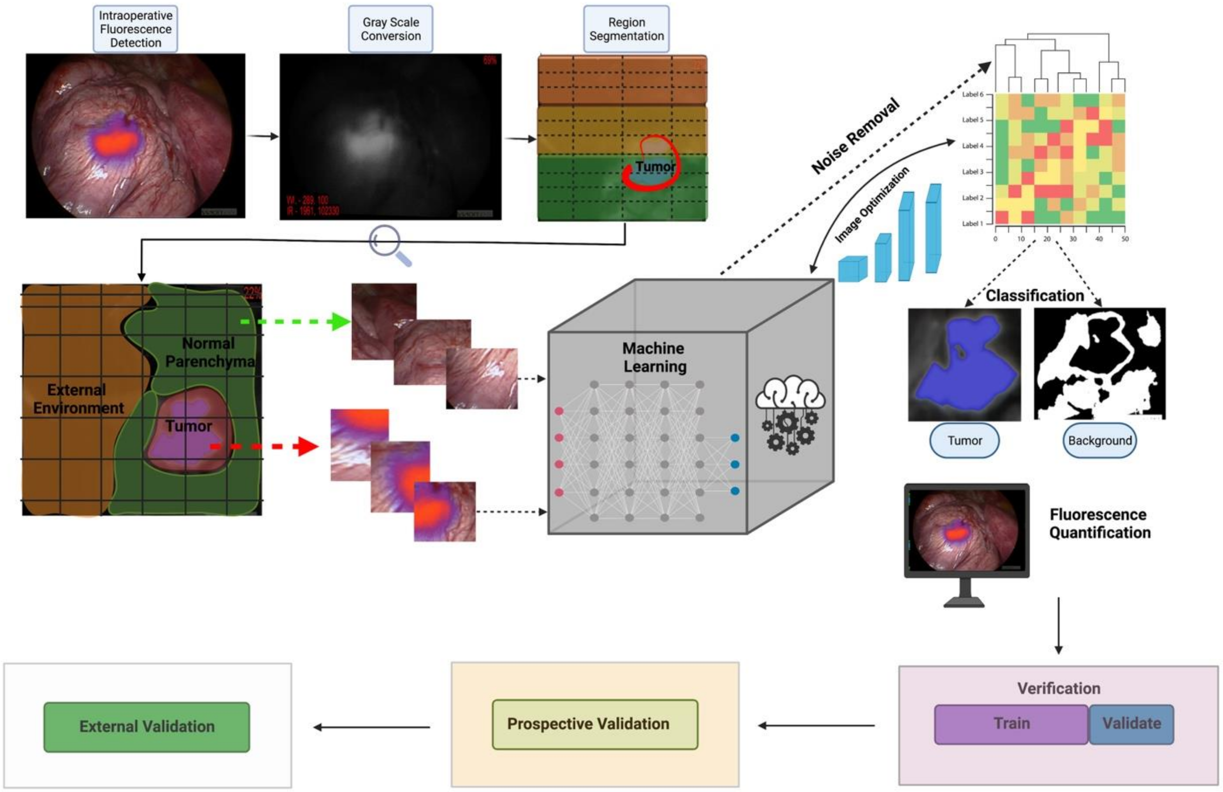

eFigure 7. Image Analysis Protocol

Correct image analysis and fluorescence quantification are critical for the nomogram's optimal predictive ability. To generate an objective calculation, images containing a minimum of 4651 pixels per frame were converted into NIR grayscale images, and each pixel was then segmented. Segmented areas of interest, including tumor/nodule, normal parenchyma, and external environment, were then classified into MATLAB Simulink R2022b software. The software was then used to calibrate individual frames and remove noise, which was then added into ML to generate appropriate classification of the tumor and background. The system then generated the fluorescence intensity of the tumor and background and ultimately calculated TBR values. The algorithm was then validated in the validation cohort and prospectively integrated into IMI-guided lung cancer resection clinical studies.

© 2026 Azari F et al. *JAMA Network Open*.

eAppendix.

Automatic TBR Calculation based on Tumor and Background area detection using image segmentation

Algorithmic TBR detection was implemented in Matlab R2020b using Image Processing Toolkit, advanced image recognition function with the original algorithm developed by authors.

Sample input image was in jpg format shown in S-2.

As a first step, image segmentation was performed and tumor area was extracted, image file was converted to the high-contrast binary format to be used as a mask (S-3).

Next, mask was applied to the original image using bsxfun function (S-4). Below are two calls of bsxfun, first extracts the tumor area, second call extracts rest of the image.

```
maskedRgbImage = bsxfun(@times, grayscaleImage, cast(tumorMask,
'like', grayscaleImage)
figure, imshow(maskedRgbImage)

backgroundImage = bsxfun(@times, grayscaleImage, cast(~tumorMask,
'like', grayscaleImage)
```

S-5. Shows standalone tumor area extracted from original grayscale image. Gray color spectrum is 0 to 255, 0 corresponds to black color.

The extraction of values from image as a numeric array was trivial at this point, thanks to Matlab's built-in features:

```
tumorPixelValues = grayscaleImage(tumorMask);
```

The image segmentation approach (but with different object detection) was repeated second time. This time it was applied to the original image with tumor area extracted (S-6). The detected full organ as a mask is shown in S-7. After the noise reduction, better version of the mask is shown in S-8. Noise reduction was done by performing gaussian smoothing followed by calculating Sobel and Prewitts gradient operators. Details are omitted here since method are widely cited in the literature [ref].

© 2026 Azari F et al. *JAMA Network Open*.

As shown in S-8, there are still some visual artefacts present in the image even after extensive noise reduction processing was applied to it. This signifies the usage of better-quality image for achieving more precise TBR measurement.

To extract pixel values for the full but minus the tumor area (to avoid double calculation for the tumor area) – a combined mask was constructed by superimposing the organ and tumor areas (S-9).

Finally, numeric values from image objects were extracted and TBR calculated:

```
backgroundPixelValues = grayscaleImage(combinedBkgMask);

tumorIntensity = mean(tumorPixelValues, 'all')

backgroundIntensity = mean(backgroundPixelValues,'all')

  TBR = tumorIntensity/backgroundIntensity
```

For practical implementation algorithm was compiled by Matlab compiler as a standalone Excel Add-On module for Windows and as a command-line tool for Windows and Mac OS X platforms. As an input the tool accepts an image file name and outputs TBR numeric value.

Authors continue working on implementing the algorithm as a pure C/C++ code to be used for embedded systems, such as cameras.

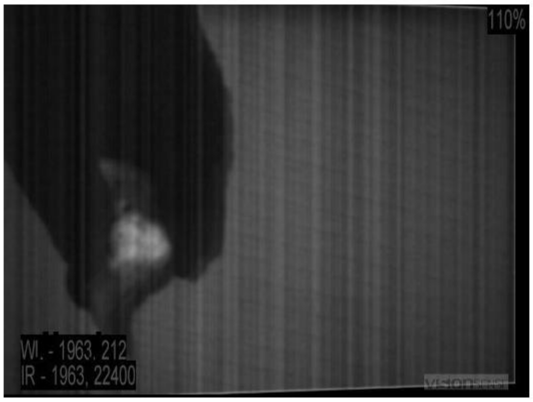

S-2. Original NIR Image

© 2026 Azari F et al. *JAMA Network Open*.

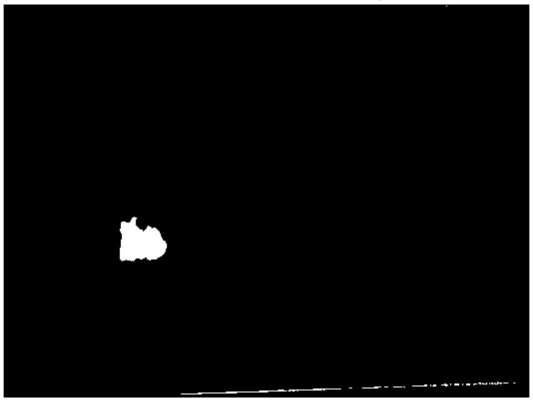

S-3. Extracted tumor area, converted to B/W mask.

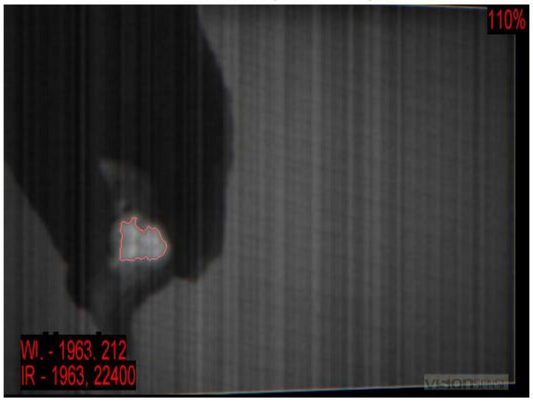

S-4 Original B/W image with applied tumor mask overlay.

© 2026 Azari F et al. *JAMA Network Open*.

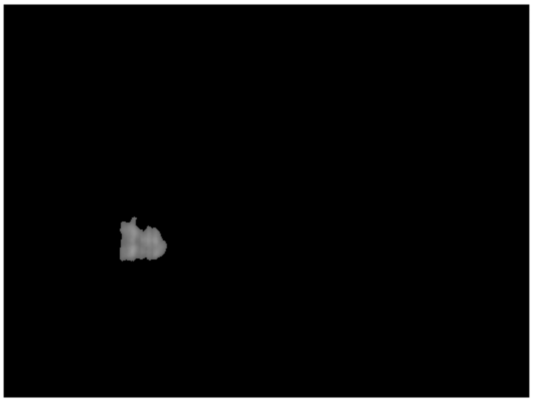

S-5. Extracted tumor area in grayscale format.

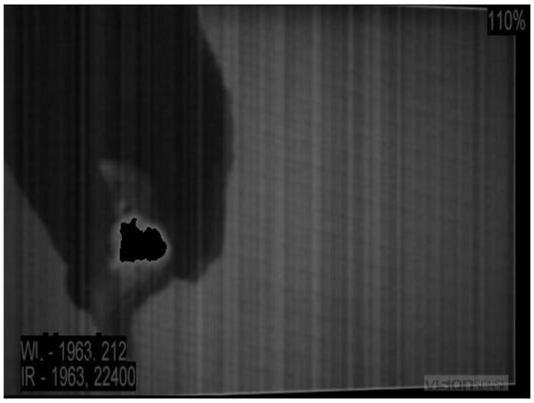

S-6. Original image with tumor area extracted.

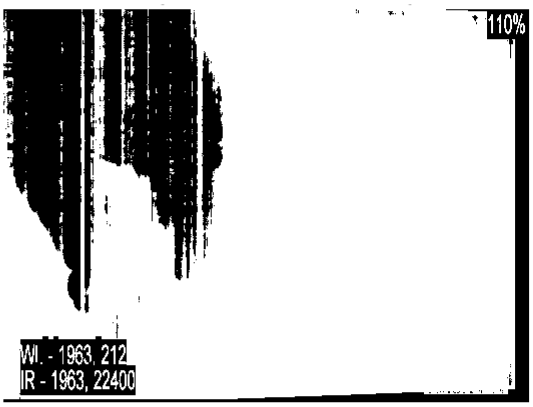

© 2026 Azari F et al. *JAMA Network Open*.

S-7. Full organ detection.

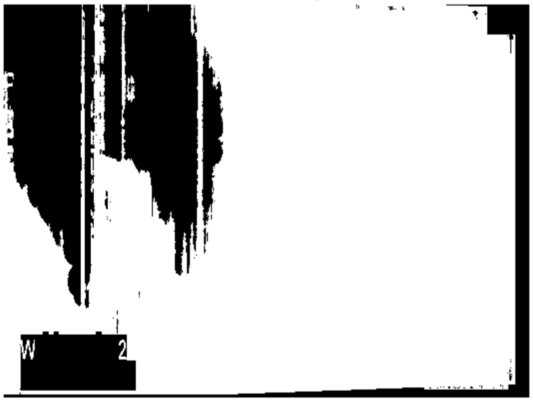

S-8. Full organ detection with noise reduction applied.

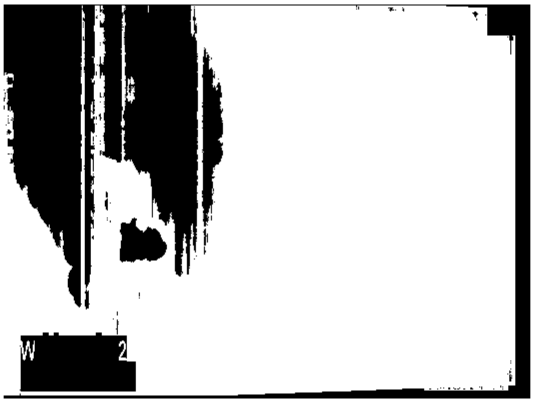

S-9: Combined Organ and Tumor mask.

eReference

https://www.mathworks.com/help/images/reduce-noise-in-image-gradients.html

© 2026 Azari F et al. *JAMA Network Open*.
