# Supplementary material for: Machine Learning–Guided Detection of Malignancy of Lung Nodules With Molecular Imaging–Guided Surgery
Source: JAMA Netw Open. 2026 Jan 13;9(1):e2551734. doi: 10.1001/jamanetworkopen.2025.51734 (PMC12801086; doi:10.1001/jamanetworkopen.2025.51734)
Supplement: Supplement 2. — Data Sharing Statement [file jamanetwopen-e2551734-s002.pdf]

Data Sharing Statement

Azari. Machine Learning–Guided Detection of Malignancy of Lung Nodules With Molecular Imaging–Guided Surgery. *JAMA Netw Open*. Published January 08, 2026. doi:10.1001/jamanetworkopen.2025.51734

Data
Data available: No

Additional Information
Explanation for why data not available: Data Availability: The main data supporting the results in this study are available within the paper and its Supplementary Information. Additional data are not publicly available because they are electronic health records, consented for research use by Penn Medicine investigators. Making the data publicly available without additional consent or ethical approval might compromise patients' privacy and the original ethical approval. If other investigators are interested in performing additional analyses, requests can be made to the corresponding author (S.S) and analyses will be performed in collaboration with Penn Medicine and the University of Pennsylvania. Code Availability: The ML algorithm cannot be made publicly available because it is proprietary intellectual property (patent pending). The AI algorithm cannot be used in routine practice before obtaining FDA approval, and this algorithm is currently undergoing a submission/review process with the USPTO (US Provisional Patent Application No. 63/291,179). The ML algorithm is available upon request for research studies.
